# Naphthalene-based fluorescent probes for glutathione and their applications in living cells and patients with sepsis: Erratum

**DOI:** 10.7150/thno.107439

**Published:** 2024-12-01

**Authors:** Jun Li, Younghee Kwon, Kyung Soo Chung, Chang Su Lim, Dayoung Lee, Yongkang Yue, Jisoo Yoon, Gyoungmi Kim, Sang-Jip Nam, Youn Wook Chung, Hwan Myung Kim, Caixia Yin, Ji-Hwan Ryu, Juyoung Yoon

**Affiliations:** 1 Department of Chemistry and Nano Science, Ewha Womans University, Seoul 120-750, Korea; 2 Severance Biomedical Science Institute, Brain Korea 21 PLUS Project for Medical Science, Yonsei University College of Medicine, Seoul 120-752, Korea.; 3 Division of Pulmonology, Department of Internal Medicine, Institute of Chest Disease, Severance Hospital, Yonsei University College of Medicine, Seoul 120-752, Korea.; 4 Key Laboratory of Chemical Biology and Molecular Engineering of Ministry of Education, Key Laboratory of Materials for Energy Conversion and Storage of Shanxi Province, Institute of Molecular Science, Shanxi University, Taiyuan 030-006, China.; 5 Department of Chemistry and Energy Systems Research, Ajou University, 443-749, Suwon, Korea.

The authors apologize that the original version of this paper unfortunately contained an incorrect **Figure 9** which is the same as **Figure 8**, due to our carelessness in proofreading. We must correct **Figure 9** as below:

## Figures and Tables

**Figure 9:**
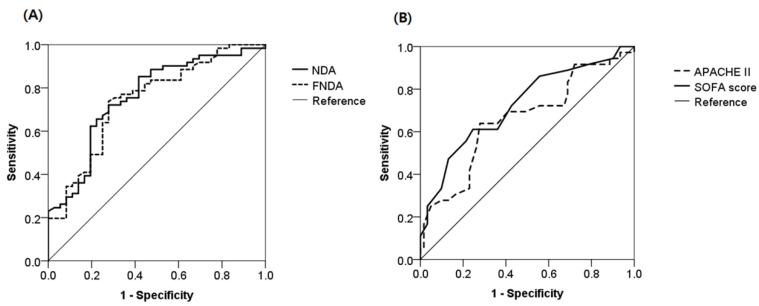
ROC curves used to predict mortality in sepsis patients. (A) ROC curves of NDA and FNDA. (B) ROC curves of APACHE II and SOFA scores.

